# Perspectives on Collaboration between Physicians and Nurse Practitioners in Japan: A Cross-Sectional Study

**DOI:** 10.3390/nursrep12040086

**Published:** 2022-11-21

**Authors:** Mari Igarashi, Ryuichi Ohta, Akinori Nakata, Yasuo Kurita, Yuta Mitobe, Miho Hayakawa, Tsutomu Yamazaki, Harumi Gomi

**Affiliations:** 1 Graduate School of Medicine, International University of Health and Welfare, Tokyo 107-8402, Japan; 2 Graduate School of Health and Welfare Sciences, International University of Health and Welfare, Tokyo 107-8402, Japan; 3 Community Care, Unnan City Hospital, Unnan 699-1221, Japan; 4 School of Medicine, International University of Health and Welfare, Narita 286-8686, Japan

**Keywords:** Japanese nurse practitioner, collaboration, perspectives

## Abstract

Background: Nurse practitioners (NPs) are known as effective healthcare providers worldwide. In Japan, nurse practitioner adoption is considered to be in a shaky period. Although nurse practitioners were introduced approximately 10 years ago at the initiative of educational institutions in Japan, the full extent of this trend is not known. Therefore, we have clarified the whole picture of nurse practitioners from two directions: the perception of nurse practitioners in Japan and the perception of physicians who work with nurse practitioners. This will inform discussions regarding the recruitment of nurse practitioners at the national level in Japan. Methods: From 18 June to 24 July 2021, we administered a nationwide cross-sectional survey of NPs and physicians working in the same clinical settings as NPs in Japan. The domains of the survey included “scope and content of work”, “perceptions of NPs’ clinical practice”, and “individual clinical practice characteristics”. The survey was distributed and collected digitally. Results: The total number of respondents to the survey was 281, including 169 NPs and 112 physicians; the percentage of NPs who responded was 50.5%. The number of valid responses was 164 NPs and 111 physicians, for a total of 275 respondents. Approximately 60% of NPs are concentrated in Tokyo, the capital of Japan, and the three prefectures adjacent to Tokyo. They also worked fewer hours per week, cared for fewer patients per day, and earned less money than physicians. More physicians than NPs indicated that “more NPs would improve the quality of care”. A total of 90.1% of physicians and 82.3% of NPs agreed that “Nurse practitioners should practice to the full extent of their education and training,” and 73.9% of physicians and 81.7% of NPs agreed that “Nurse practitioners’ scope of practice should be uniformly defined at a national level”. Conclusions: This study clarified the present working conditions of NPs from NPs’ and physicians’ perspectives in Japanese contexts. Japanese NPs may be able to work effectively in collaboration with physicians. Therefore, the implementation of NPs in Japanese medical conditions should be discussed further for better healthcare.

## 1. Introduction

Nurse practitioners (NPs) are known as healthcare providers who contribute to improving access to healthcare and patient satisfaction [1,2,3]. The recruitment of NPs for health care innovation in many countries has become a global trend [4]. International standards for NPs were set forth by the International Council of Nurses in 2020, but specific authority and job descriptions vary depending on the employing country [5]. The United States, where most NPs practice independently, has been active for over 50 years since the birth of NPs, making them indispensable, especially as primary care providers [6]. Perhaps because of this, many NPs in the U.S. recognize that their practice improves the safety, efficiency, etc., of medical care, but this does not necessarily mean that U.S. physicians have the same perception as NPs [7]. The evaluation of NP practices by physicians, who are the primary users of medicine, influences healthcare policy decisions.

In Japan, on the other hand, NPs were created approximately 10 years ago, following the model of NPs in the United States. Japanese NPs are not certified by the national government but by an organization composed of graduate schools that train NPs [8]. Japan’s medical background is a country that has adopted the universal health insurance system recommended by the World Health Organization, a system in which anyone can receive medical care anywhere at a low cost, and a long-term care insurance system that covers care for the elderly throughout the country. However, the country continues to have the largest proportion of elderly people in the world, and the declining birthrate is not slowing down, so the question is whether this system can be maintained [9,10,11]. As part of its efforts to maintain the healthcare delivery system, the Japanese government is steering the transfer of physicians’ duties to non-physician healthcare professionals. In particular, a system was created in 2015 for nurses to be able to perform 38 specific types of medical procedures under comprehensive instructions from physicians if they receive training at institutions designated by the government [12]. Originally, the law stipulated that the duties of Japanese nurses were to “care for the medical treatment of patients” and “assistance in the treatment of physicians” [13]. The Specific Medical Practice training system has positioned nurses’ medical practice as “assisting physicians in the practice of medicine”, and the organization that oversees graduate schools that educate NPs has mandated training in specific medical practices as part of their educational curriculum.

On the other hand, the process of this legalization led to the interpretation that the scope of duties of nurses would be limited to certain medical procedures and that nurses could perform even relatively invasive medical procedures, such as intubation and extubation, as “assisting physicians” if directly instructed by the physician.

Therefore, at present, NPs in Japan practice the medical acts specified by the government under the comprehensive supervision of physicians, and practice other medical acts under the direct supervision of physicians within the scope of their discretion.

Given these factors, it is extremely important to know how physicians evaluate NPs in their clinical practice in order for NPs to operate in Japan. Therefore, this study sought to clarify the current status of NPs’ job descriptions in Japan and to determine how NPs and physicians who work with NPs perceive the current status of NPs.

This is the first report of its kind in East Asian countries. Therefore, the clarification of these findings may provide significant data for discussions on the official use of NPs in Japan in the future, and may influence decision-making on the introduction of NPs in countries in the midst of the NP adoption wave, especially in East Asian countries with similar cultural backgrounds.

## 2. Materials and Methods

### 2.1. Study Design

This study is a national cross-sectional survey of NPs and physicians collaborating with NPs in Japan; collaborating with an NP was defined as working in the same department. We conducted this survey online from 18 June to 24 July 2021.

### 2.2. Samples

The sampling of NPs in Japan was 338 of the 583 NPs whose credentials had been certified by the Japanese Organization of Nurse Practitioner Faculties (JONPF) by 31 March 2021, and who had given permission to be contacted for research purposes. The sampling of physicians working with the NPs included the physicians in their departments. The NPs were asked to distribute the questionnaire to the relevant NPs via email from JONPF, and we asked NPs to distribute questionnaires to the physicians who collaborate with them. Because physicians were asked to participate in this survey via NPs by the snowball method, we did not count the number of questionnaires distributed to physicians. There-fore, we could not tell the response rate for physicians.

### 2.3. Measurements

The questionnaire was developed by Donelan [14] in 2020 and modified for the Japanese version after obtaining permission from the authors. The modifications were made by having five experts with knowledge of the medical backgrounds of the U.S. and Japan and familiar with the activities of NPs in Japan validate the questionnaire from previous studies and modify it to fit the actual situation in Japan. The domains of the questionnaire included the “scope and content of work.”, “perceptions of NPs’ clinical practice.” and “individual clinical practice characteristics.”. The questionnaires were distributed and collected via the Internet using Google Form^TM^.

### 2.4. Analysis

The response data were corrected for suspected outliers by confirming the correct values with the respondents via e-mail. Statistical analysis was conducted for the two groups of NPs and physicians working with NPs, with a significance level of 0.05 (95% confidence interval), and logistic regression analysis was performed. SPSS Statistics version 27 (IBM, New York, NY, USA) [15], and EZR (Saitama Medical Center, Jichi Medical University, Saitama, Japan) were used for statistical analysis. The χ-square test was used for variables on the nominal scale, and Fisher’s exact test was used for those with an expected frequency of less than 5. For scale variables, *T*-tests or Mann–Whitney U-tests were used, depending on the characteristics of the data.

### 2.5. Ethical Considerations

The participation of the research collaborators in the questionnaire clearly stated that they were deemed to have given their consent by answering the questionnaire so that the free will of the individuals could be respected. In addition, participant information obtained from the questionnaire responses was not used for any purpose other than research purposes and was kept in strict confidence so as not to be leaked. The contact information for the respondents who wished to withdraw their responses was indicated, and it was guaranteed that they could withdraw their responses before the publication of the research results. Although respondents were free to write their names on the questionnaire, we required them to provide their e-mail addresses so that we could confirm their answers if they were clearly erroneous. The study was conducted with the approval of the International University of Health and Welfare Ethics Committee. The ethics approval number is 20-Im-017. Our study was reported according to the Strengthening the Reporting of Observational Studies in Epidemiology (STROBE) guidelines.

## 3. Results

The total number of respondents to the survey was 281, including 169 NPs and 112 physicians; the percentage of NPs responding was 50.5%. The numbers of valid responses were 164 NPs and 111 physicians, for a total of 275 respondents.

### 3.1. Characteristics of Respondents

Regarding the characteristics of the respondents (Table 1), in terms of gender, the NPs were predominantly female (59%) and the physicians were predominantly male (94%) (*p* < 0.001). The mean age was younger for NPs; it was 37.4 years (SD 22.1) for NPs and 45.2 years (SD 21.9) for physicians. In terms of final education, all NPs had a master’s degree or higher, and 0.6% had a doctor’s degree. Among the physicians, 10.2% had a master’s degree and 27.0% had a doctorate. Since annual income was not required question, there were 207 respondents; 80.3% of NPs had annual incomes between JPY 5 and 10 million, while 88.2% of physicians had annual incomes of JPY 10 million or more, a significant difference (*p* < 0.001). There was a significant difference in the mean number of years of clinical experience, with 18.9 years (SD 10.2) for physicians and 3.59 years (SD 3.2) for NPs (*p* < 0.001). Among them, the Tokyo metropolitan area (Tokyo and the three prefectures adjacent to Tokyo: Kanagawa, Saitama, and Chiba) accounted for about 60%. The largest number of both physicians and NPs belonged to hospitals with fewer than 20–500 beds (60%). The most common affiliation for both physicians and NPs was the emergency department (MD 15.3%, NP 16.5%, *p* = 0.779), followed by general practice medicine (MD 12.6%, NP 9.8%, *p* = 0.456), and then cardiovascular surgery (MD 7.2%, NP 7.9%, *p* = 0.826). The number of actual hours worked per week was significantly different for NPs, averaging 50.3 h versus 58.6 h for physicians (*p* < 0.001); the number of patients cared for per day was significantly different for NPs, with 10.5, versus 19.4 for physicians (*p* < 0.001).

### 3.2. NPs in Clinical Practice in Japan

Table 2 shows the actual job description of NPs: the most common response by the NPs themselves was “blood sampling by arterial puncture” (86.6%), followed by “history taking and physical examination” (76.8%), and the third was “interpretation of ECGs” (75.0%). The most common response on the physician’s side was “history taking and physical examination” (67.8%), followed by “peripheral indwelling central venous catheter (PICC) insertion” (62.2%), and the third was “interpretation of ECG” (61.3%). Those that were answered by more than 60% of the physicians and NPs and did not differ significantly were “history taking and physical examination” (*p* = 0.089), “PICC insertion” (*p* = 0.676), and “performing a simple ultrasound examination” (*p* = 0.144). Regarding whether the impact of the spread of the novel coronavirus disease 2019 (COVID-19) infection changed NPs’ job descriptions, 38.4% of the NPs and 31.5% of the physicians reported that they had changed, with a non-significant difference. In the open-ended responses on how it changed, negative responses indicated that regular medical care was not provided, while positive responses indicated that the importance of NPs was made known in the hospital through their special duties on the front lines of infectious diseases and their full-time intensive care of patients with severe coronary disease.

### 3.3. Perception of the Team

In terms of the perceptions about the team (Table 3), for the question “Who are the team members you work with every day?”, the most common responses from physicians were, in descending order, 90.1% nonresident physicians, 94.6% registered nurses, 82.0% NPs, 63.1% residents and pharmacists, 60.4% physical therapists, and 54.1% medical social workers. In the advanced practice nursing field other than NPs, 14.4% were professional nurses, 25.2% were certified nurses, and 1.8% were nurses who had completed specific practice training.

The occupations with significant differences between the NP and physician responses were NPs (32.9%, *p* < 0.001), non-NP nurses who had completed specific practice training (16.5%, *p* < 0.001), and certified nurses (37.8%, *p* = 0.029).

In response to the statement “When physicians and NPs perform the same types of procedures and laboratory tests, physicians provide higher quality care than NPs”. Approximately 36% of both physicians and NPs agreed with the statement. In addition, 76.6% of physicians and 59.1% of NPs (*p* = 0.003) agreed with the statement “The physicians I work with trust the skills and clinical judgment (decision making) of NPs”. For the statement “NPs are effective leaders of the care team, which includes physicians, nurses, and other health professionals”, 55.0% of physicians and 39.0% of NPs were in agreement.

### 3.4. Respondents’ Views on the Effect of an Increased Supply of Nurse Practitioners on the Quality of Healthcare

Regarding the question about improving the quality of care (Table 4) by increasing the number of NPs, a total of 81.1% of physicians and 71.3% of NPs agreed that “safety will improve”, while 88.3% of physicians and 84.1% of NPs agreed that “timeliness will improve”. Additionally, 88.3% of physicians and 84.1% of NPs agreed with “better timeliness” 78.4% of physicians and 72.0% of NPs agreed with “better effectiveness”, and 73.0% of physicians and 71.3% of NPs agreed with “better patient-centeredness”. All responses were related to improving the quality of care with more NPs. The percentage of physicians who responded “better.” on all items was higher than that of NPs. Items with significant differences were cost-effectiveness (84.7% of physicians vs. 65.9% of NPs, *p* < 0.001) and patient clinical outcomes (68.5% of physicians vs. 50.0% of NPs, *p* = 0.002).

### 3.5. Perceptions of NP Policy and Practice in Japan

Regarding the perceptions of NP policy and practice (Table 5), 90.1% of physicians and 82.3% of NPs agreed with the statement “NPs should practice the full range of their education and training”. Total of 73.9% of physicians and 81.7% of NPs agreed with the statement “The scope of practice of NPs should be uniformly defined at the national level”, and 28.9% of physicians and 28.7% of NPs agreed with the statement “The physicians I work with do not understand NPs”. In contrast, approximately 70% of both physicians and NPs disagreed with the statement “The physicians I work with do not understand NPs”. There was a significant difference for the statement “Physicians and NPs need to be paid the same fees to provide or perform the same services and procedures”, with 36.9% of physicians and 54.3% of NPs in agreement (*p* = 0.005).

## 4. Discussion

A cross-sectional survey administered at the same time for two target groups, NPs and physicians working with NPs, revealed the current status of NPs in Japan and differences in the perceptions between the two groups. In addition, the questionnaire was modified from the one administered in the U.S. to the Japanese version, so many of the responses could be compared with those in the U.S. The results of the survey were also compared with those in the U.S. Although the two groups cannot be compared in exactly the same way due to differences in the time period, social background, and sampling of the study subjects, we compared the perceptions between Japanese physicians and NPs from various perspectives, including similarities and differences.

One characteristic of Japanese NPs was that many of them were engaged in critical care in the Tokyo metropolitan area and other urban centers. This indicates that, unlike in the U.S. and other countries, NPs did not have an impact as a presence to meet the demand for medical care in medically underpopulated areas where there were no physicians.

In terms of the gender ratio of respondents, similar to the NP group in the 2020 survey in the U.S. [14], there were more women in the NP group than in the physician group, but the difference was that the proportion of men in the NP group was twice that in the U.S. The NP group was more likely to be male than female. Since this is a phenomenon not seen internationally, this may be a new model for discussing gender differences between men and women.

There is clearly a difference in annual income between physicians and NPs. However, this may reflect the nature of their practice due to differences in the number of hours worked per week and the number of patients cared for per day, as well as differences in the number of years of clinical experience with NPs, gender differences, and age differences. Japan has an inherent seniority system in which salaries increase with age. Many physicians did not require NPs to be on-call, etc., and perceived that since the responsibility is solely on the physician, the income would not be the same.

As for the specific job description of NPs, it was recognized that their job was to take the patient’s history and perform physical examinations. In addition, non-invasive examinations, such as electrocardiograms and simple ultrasound examinations, and invasive but minor arterial blood sampling for blood gas analysis were frequently performed, and this indicates that many NPs in Japan are trying to obtain physical information on patients in critical situations by using medical knowledge and technology. Furthermore, in device-related procedures, Japanese national specific acts were performed more than non-specific acts, and PICC insertion, among others, was recognized as an act that symbolizes NPs. Approximately 36% of both physicians and NPs believed that “physicians provide higher quality care than NPs when they perform the same type of procedure or perform a clinical examination.” Paradoxically, this can be interpreted to mean that approximately 60% of physicians and NPs rated examinations and procedures as comparable to physician practice. In addition, 76.6% of physicians indicated that they trust the skills and clinical judgment (decision-making) of NPs, indicating that NPs receive a certain amount of positive feedback on their medical thinking and skills from the physicians they work with. In terms of perceptions within the team, 82% of physicians perceived that they always work with NPs and only 1.8% of non-NP nurses who have completed specific practice training. The fact that 32.9% of NPs but only 1.8% of physicians recognized those who had completed specific act training indicates that physicians do not recognize them as part of the team.

In fact, as of June 2021, when this survey was conducted, the actual number of nurses who had completed the specific act was 3307; subtracting the 583 NPs, the number was 2724, which is 4.67 times the number of NPs. This suggests that the government wants nurses who have completed specific practice training to function as key players in team medicine, but in order to do so, they will first need to be recognized as part of the team. Regarding the NP’s leadership within the team, 55% of physicians agreed with the statement “The NP is an effective leader of the care team, which includes physicians, nurses, and other health professionals”, indicating that more than half of the physicians in the field working with NPs rated NPs as functioning as team leaders.

In this study, NPs were sampled from the entire population. Therefore, one might argue that this is why it deserves to be a recommendation for national policy. On the other hand, the sampling of physicians was purposive and does not reflect the opinions of the population of physicians in Japan as a whole. However, at the very least, physicians who have seen NPs up close in actual clinical practice will better understand their capabilities than physicians who do not know them. They would be in a position to evaluate safety concerns even more severely since the physician who issued the order would be held accountable. Thus, this sampling provided a deep, multifaceted, and quantitative picture of the current status of NPs in real-world clinical practice. The results that many of these physicians recognize that increasing the number of NPs will improve the quality of medical care, that they practice the full range of education and training, and that the government should define the scope of their practice should serve as a reference for policy makers as they work to reform Japan’s healthcare delivery system.

On the other hand, however, it is puzzling that NPs are less likely than physicians to believe that they themselves are contributing to improving the quality of medical care. Japan has the virtue of “modesty” and Japanese NPs believe in modesty [16]. This is a phenomenon that is difficult for Westerners to understand, often likened to a “bamboo ceiling”, and understood as a negative in career development in the international community, especially in the West. However, in Japan, it is considered wisdom for career development without causing friction in society [17]. If this is the cause, there is no need to see it as a problem when practiced in the Japanese context. On the other hand, if it is due to a lack of clinical experience, then it will change over time, and no special measures will be necessary. If, however, the cause is that NPs feel incompetent and lack confidence, then additional education to build competence or an improved educational system may be necessary. In any case, the cause of this problem needs to be clarified in the future. This is because if NPs are to acquire prescriptive rights and assume independent practice in the future, it is a prerequisite that they demonstrate their own competence to those around them.

### Limitation

The sampling of physicians in this survey is purposive sampling, and physicians who are not favorable to NPs may not have responded. Therefore, the opinions cannot be said to be representative of physicians throughout Japan, nor can a simple comparison of Japan and the U.S. be made. In the future, a nationwide survey with a randomized sampling of physicians is needed as the number of NPs expands.

In addition, this survey was conducted one year after the novel coronavirus disease 2019 began to spread in Japan in 2020, which can be considered a period of change in which normal medical care was often not provided, which may have affected the results of the survey in some way. It should be also noted that healthcare systems are different by countries and cultures, suggesting that these factors should be considered in the future studies [18]. In the future, it will be necessary to continue to investigate their duties and to further investigate their role in the medical care that is being provided along with COVID-19 infection.

## 5. Conclusions

This study clarity the present working conditions of NPs from NPs’ and physicians’ perspectives in Japanese contexts. Japanese NPs may be able to work effectively with the collaboration with physicians. Therefore, the implementation of NPs in Japanese medical conditions should be discussed further for better healthcare.

## Figures and Tables

**Table 1:** Characteristics of Respondents.

Total N = 275		MD		NP		*p*-Values
		111		164		
Gender	Male	105	94.0%	67	40.0%	<0.001
	Female	6	5.0%	97	59.0%	
Age	<45	49	44.1%	100	61.0%	0.006
	45+	62	55.9%	64	39.0%	
	average	45.2	SD: 21.9	37.4	SD: 22.1	0.012
Last educational background	Bachelor	69	62.2%	0		<0.001
	Masters	12	10.2%	163	99.4%	
	Doctorate	30	27.0%	1	0.6%	
Income (JPY) * N = 207	<5,000,000	3	3.5%	23	18.9%	<0.001
	5,000,000~9,990,000	7	8.2%	98	80.3%	
	10,000,000+	75	88.2%	1	0.8%	
Years in practice (average)		18.9	SD:10.2	3.5	SD:3.2	<0.001
***Practice Characteristics***						
Hospital Size (Beds)	<20	7	6.3%	4	2.4%	0.314
	20~499	66	59.5%	101	61.4%	
	500+	36	32.4%	58	35.4%	
Work area (prefecture)	Tokyo, the capital of Japan, and the three prefectures adjacent to Tokyo	67	60.3%	97	59.10%	0.84
	Otherwise	44	39.6%	67	40.8%	
Department	**Clinical Physician Context**	**110**	**99.0%**	**143**	**87.1%**	
	Emergency Medicine	17	15.3%	27	16.5%	0.779
	General internal medicine	14	12.6%	16	9.8%	0.456
	Cardiovascular Surgery	8	7.2%	13	7.9%	0.826
	**Otherwise**	**1**	**0.9%**	**21**	**12.8%**	
	Home Nursing Station	0	0.0%	1	0.6%	<0.001
	Other	1	0.9%	20	12.1%	<0.001
Actual hours per week (average)		58.6	SD:18.1	50.3	SD:18.1	<0.001
Number of patients per day (average)		19.4	SD:28.1	10.52	SD:14.1	<0.001

MD: Medical Doctor, NP: Nurse Practitioner. SD: Standard Deviation. * JPY: Japanese Yen (legal tender).

**Table 2:** NPs in Clinical Practice in Japan.

Total N = 275	MD		NP		*p*-Values
	111		164		
***In my department, NPs***					
Take history and perform physical examinations	75	67.8%	126	76.8%	0.089
Develop and implement treatment and care plans for the management of acute illnesses	37	33.3%	82	50.0%	0.006
Proposes and interprets results of laboratory studies	64	57.7%	119	72.6%	0.01
Consults with Experts	46	41.4%	84	51.2%	0.111
Suggests appropriate medication prescriptions	44	39.6%	99	60.4%	<0.001
Explains procedure (necessity, preparation, nature, and effects) to patients, patient’s families	47	42.3%	90	54.9%	0.041
Works with patients and families on palliative care and end of life planning	57	51.4%	95	57.9%	0.282
Performs spinal or joint taps	22	19.8%	29	17.7%	0.655
Performs basic procedures for wounds and abscesses (sutures, debridement, drain ulcers)	53	47.7%	91	55.5%	0.207
Performs intubation	28	23.4%	54	32.9%	0.089
Inserts central line (subclavian, internal jugular)	38	34.2%	58	35.4%	0.847
Leads team rounds	28	25.2%	48	29.3%	0.462
Interprets ECGs	68	61.3%	123	75.0%	0.015
Response to emergencies RRT/codes	65	58.6%	90	54.9%	0.546
On call (carries beeper) nights and weekends	12	10.8%	32	19.5%	0.053
Pleural and ascites puncture	34	30.6%	55	33.5%	0.613
Inserts PICCs (peripherally inserted central venous catheters)	69	62.2%	106	64.6%	0.676
Performs simple ultrasound examinations	67	60.4%	113	68.9%	0.144
Punctures artery and collect bloods	67	60.4%	141	86.6%	<0.001
Adjustments of ventilator settings	55	49.5%	110	67.1%	0.004
Performs extubations	46	41.4%	83	50.6%	0.135
Managements of ECMO operations	10	9.0%	32	19.5%	0.018
Management of dialysis and filtrations	15	13.5%	42	25.6%	0.015
Performs surgical assists in operating room	38	34.2%	80	48.8%	0.017
NP’s job description has changed in the COVID-19 pandemic	35	31.5%	63	38.4%	0.242

**Table 3:** Perceptions of the team.

Total N = 275	MD		NP		*p*-Values
	111		164		
***Who do you work with on a daily basis?***					
Registered nurse	105	94.6%	142	86.6%	0.310
Nurse Practitioner	91	82.0%	54	32.9%	<0.001
Certified Nurse Specialist	16	14.4%	24	14.6%	0.960
Nurse (other than NP) who have completed Specific Practice Training	2	1.8%	27	16.5%	<0.001
Certified Nurse	28	25.2%	62	37.8%	0.029
Physician	100	90.1%	152	92.7%	0.446
Resident	70	63.1%	108	65.9%	0.635
Pharmacist	70	63.1%	91	55.5%	0.211
Physical Therapist	67	60.4%	87	53.0%	0.231
Occupational Therapists	46	41.4%	66	40.2%	0.843
Clinical Engineer	43	42.7%	70	42.7%	0.514
Medical Social Worker	60	54.1%	68	41.5%	0.040
Care Manager	14	12.6%	21	12.8%	0.963
Care Worker	10	9.0%	11	6.7%	0.481
When physicians and nurse practitioners perform the same type of procedure or clinical examination physicians provides higher quality care than nurse practitioners	41	36.9%	60	36.6%	0.953
Physicians with whom I work trust nurse practitioner’s skills and clinical decision making	85	76.6%	97	59.1%	0.003
Nurse practitioners are effective leaders of care teams that include physicians nurses and other health professionals	61	55.0%	64	39.0%	0.009

**Table 4:** Respondents’ Views on the Effect of an Increased Supply of Nurse Practitioners on the Quality of Healthcare.

Total N = 275	MD		NP		*p*-Values
	111		164		
***Make Better “strongly/somewhat agree”***					
Safety	90	81.1%	117	71.3%	0.066
Timeliness	98	88.3%	138	84.1%	0.334
Effectiveness	87	78.4%	118	72.0%	0.23
Efficiency and cost-effectiveness	94	84.7%	108	65.9%	<0.001
Equity	58	52.3%	74	45.1%	0.246
Patient-centeredness	81	73.0%	117	71.3%	0.768
Patient clinical outcomes	76	68.5%	82	50.0%	0.002

**Table 5:** Perceptions of NP Policy and Practice in Japan.

Total N = 275	MD		NP		*p*-Values
	111		164		
***(% responding “strongly/somewhat agree”)***					
Nurse practitioners should practice to the full extent of their education and training	100	90.1%	135	82.3%	0.073
Physicians and nurse practitioners should be paid the same fees for providing or performing the same services and procedures	41	36.9%	89	54.3%	0.005
Full-time nurse practitioners should be required to work the same hours (including shifts and on call coverage) as full-time physicians	35	31.5%	67	40.9%	0.116
Nurse practitioners’ scope of practice should be uniformly defined at a national level	82	73.9%	134	81.7%	0.121
The physicians with whom I work do not understand nurse practitioners education and training	32	28.9%	47	28.7%	0.976

## Data Availability

All relevant datasets in this study are described in the manuscript.
